# WISP-1 Contributes to Fractionated Irradiation-Induced Radioresistance in Esophageal Carcinoma Cell Lines and Mice

**DOI:** 10.1371/journal.pone.0094751

**Published:** 2014-04-11

**Authors:** Wen-Feng Li, Li Zhang, Hai-Ying Li, Si-Si Zheng, Liang Zhao

**Affiliations:** 1 Department of Radiation Oncology, the First Affiliated Hospital of Wenzhou Medical University, Wenzhou, Zhejiang, China; 2 Laboratory of Internal Medicine, the First Affiliated Hospital of Wenzhou Medical University, Wenzhou, Zhejiang, China; 3 Division of PET/CT, the First Affiliated Hospital of Wenzhou Medical University, Wenzhou, Zhejiang, China; Vanderbilt University, United States of America

## Abstract

Cancer cells that survive fractionated irradiation can be radioresistant and cause tumor recurrence. However, the molecular mechanisms underlying the development of radioresistance in cancer cells remain elusive. The aim of this study was to investigate the role of WISP-1 in the development of radioresistance in esophageal carcinoma during fractionated irradiation. Radioresistant esophageal cancer cells were generated from normal esophageal cancer cells via fractionated irradiation, and expression levels of related proteins were determined by Western blot. Radiosensitivity of cells was established by clonogenic cell survival assays, and cell cycle distribution was evaluated by flow cytometry. Protein distributions were determined by immunofluorescence, and cell toxicity was evaluated by cell counting kit-8 assays. *In vivo* validations were performed in a xenograft transplantation mouse model. Our data indicate that WISP-1 plays an important role in the development of radioresistance in esophageal cancer cells during fractionated irradiation. The overexression of WISP-1 in esophageal cancer cells was associated with radioresistance. Depletion of extracellular WISP-1 by antibody neutralizing reversed radioresistance and directly induced mitotic catastrophe resulting in cell death. WISP-1 may be a candidate therapeutic target in the treatment of recurrent esophageal carcinoma after radiotherapy.

## Background

Esophageal carcinoma is a relatively rare form of cancer, but it is one of the most lethal malignancies worldwide. Globally, esophageal carcinoma causes an estimated 400,000 deaths per year [1]. There are various subtypes of esophageal carcinoma, primarily squamous cell cancer (approximately 90–95% of all esophageal cancer worldwide) and adenocarcinoma. Esophageal tumors lead to dysphagia, pain and other symptoms and are usually diagnosed by biopsy [2].

Radiotherapy is a primary treatment modality for esophageal carcinoma; however, the success of radiotherapy is limited by the presence of radioresistant cells [3]. Zhang et al. recently showed that radioresistant esophageal cancer cells can be established by repeated fractionated irradiation (FIR; the total dose of radiation is spread among fractions and delivered over time), and indicated that β-catenin might play an important role in the development of radioresistance during FIR [4]. The Wnt/β-catenin pathway can be aberrantly activated by irradiation exposure, resulting in the accumulation of β-catenin in the cytoplasm, its subsequent translocation into the nucleus, and the transcription of β-catenin target genes [5]. This aberrant activation of the Wnt/β-catenin pathway has been implicated in radioresistance of solid tumors such as glioblastoma [6], breast cancer [7], and head and neck cancer [4]. But the mechanism by which the Wnt pathway contributes to radioresistance is unclear.

WISP-1 is a member of the CCN family of growth factors and also a novel downstream target gene of β-catenin [8]. Previous studies have demonstrated that WISP-1 can attenuate p53-mediated apoptosis via the Akt pathway [9] and promote cell survival [10]. A recent study by Nagai et al. showed that expression of WISP-1 could be a clinical marker for poor prognosis in patients with esophageal squamous cell carcinoma [11]. Based on these data, we investigated the role of WISP-1 in the development of radioresistance in esophageal cancer cells during FIR.

## Methods

### Antibodies and reagents

Antibodies against total β-catenin (sc-59737; sc-7199) were purchased from Santa Cruz Biotechnology Inc. (Santa Cruz, Calif., USA); against phospho-β-catenin (Ser33/37) (2408-1), total Chk2 (3428-1), ATM (1549-1), and phospho-Chk2 (Thr68) (1538-1) were purchased from Epitomics, Inc. (Burlingame, Calif., USA); and against WISP-1 (ab10737) DNA-PKcs (ab1832), γ-H2AX (phospho-Ser139) (ab22551), β-tubulin (phosphor-Ser172) (ab76286) and β-actin (ab8226) were purchased from Abcam (Cambridge, Mass., USA). Recombinant WISP-1 protein used for extracellular stimulation was purchased from Abcam (ab50041).

### Cell culture

The normal esophageal epithelial cell line HET-1A was obtained from the American type culture collection (ATCC, Manassas, USA). The human esophageal squamous cancer cell lines KYSE-410 and TE-1 were obtained from the European Collection of Cell Cultures (Public Health England, Salisbury, UK). Cells were cultured in RPMI-1640 (Gibco, Life Technologies Inc., Grand Island, N.Y., USA) with 100 U/ml penicillin, 100 mg/ml streptomycin, 10% fetal bovine serum, and incubated at 37°C in 5% CO_2_. Radioresistant cell lines were established and cultured as reported by Zhang et al [4]. Briefly, KYSE-410 or TE-1 cells (1×10^6^) were plated in 25 cm^2^ culture flasks. Cells were irradiated with 2 Gy of 6MV X-rays (Varian 2300 C/D, USA) at a dose rate of 100 cGy per minute using a tissue compensation system with a 1.5-cm membrane. Immediately after irradiation, the culture medium was renewed, and the cells were returned to the incubator. When the cells reached approximately 90% confluence, they were trypsinized and subcultured into new flasks. When the cells reached approximately 50% confluence, they were irradiated again. The protocol included 20 fractions of irradiation at three fractions per week for 6 weeks to a total dose of 36 Gy, until radioresistant cell populations were established. Radiation-resistant clones were collected and identified as KYSE-410R and TE-1R. Individual clones were compared against a panel of clones to ensure there was no clonal variation and to confirm that identical clones could be repeatedly generated. The radioresistant cells were cultured under the conditions previously described.

### Conditional medium with high levels of secreted WISP-1

Four samples of conditioned medium with high levels of secreted WISP-1 were collected and filtered with a 0.22-µm filter from the serum-free media that had been cultured with each groups of cells for 24 h as: KYSE-410R pre-cultured (CM-KYSE-410R), TE-1R pre-cultured (CM-TE-1R), KYSE-410R pre-cultured with 3Gy irradiation (CM-KYSE-410R-3Gy) and TE-1R pre-cultured with 3Gy irradiation (CM-TE-1R-3Gy).

### Elisa assays

Samples of conditioned media were analyzed for WISP-1 concentrations by an enzyme-linked immunosorbent assay (ELISA; Quantikine, R&D Systems) according to the manufacturer's instructions. The capture antibody was diluted to an appropriate dilution in coating solution. 100 µl diluted antibody was added to the appropriate wells of a 96 well ELISA plate. The plate was then incubated for 2 hours at room temperature or 4°C overnight. The liquid was removed from the plate and the wells were washed twice with 300 µl wash solution. 300 µl blocking solution was then added to each well and the plate was incubated 1 hour. The liquid was then removed and the wells were washed twice with 300 µl wash solution. 100 µl diluted biotinylated detection antibody was then added to each well and the plate was incubated for 1 hour at 37°C or 3 hours at room temperature. The liquid was removed from the plate and each well was washed three times with wash solution. 100 µl diluted alkaline phosphatase (AKP) conjugated streptavidin was added to each well and the plate was incubated for 1 hour at room temperature. The liquid was then removed from the plate and then the wells were soaked for five minutes with wash solution. The liquid was then removed from the plate and the wells were washed with wash solution 5 times. 100 µl of the substrate para-Nitrophenylphosphate (pNPP) was then added to each well. The color was allowed to develop for 30 minutes, and then the plate was read immediately with a plate reader at 405–410 nm.

### Radiation clonogenic cell survival assay

The cells were seeded into 6-well plates at 1000 cells/well with 2 µmol/ml recombinant WISP-1 proteins or 4 µmol/ml anti-WISP-1 antibodies or conditioned medium, as appropriate. After 24 hours' exposure to WISP-1 or anti-WISP-1, the parental (KYSE-410 and TE-1) and radioresistant (KYSE-410R and TE-1R) cells were irradiated at doses of 0, 1, 2, 3, 4, 5, 6, 8, or 10 Gy at an average dose rate of 2 Gy/min. Corresponding controls were irradiated under the same conditions. Immediately following irradiation, cells were cultured for colony formation at 37°C in 5% CO_2_ for 10 days. Subsequently, colonies were fixed with pure ethanol and stained with 1% crystal violet. Control and experimental groups were run simultaneously to allow for direct comparison. Colonies containing >50 cells were counted as clonogenic survivors. The number of colonies was counted by two operators, individually. Average numbers were used for analyses to reduce counting bias. The points at 4Gy were used for statistical analysis.

### Cell cycle analysis

After 6MV γ-ray irradiation and/or treatment with WISP-1 or anti-WISP-1, cells were harvested immediately or at specified time-points post-irradiation, and fixed with 75% ethanol. Cells were re-suspended in PBS with 0.1% saponin and 1 µg/mL RNase A (Sigma-Aldrich, St. Louis, Mo., USA), incubated for 20 min at 37°C, and the DNA was stained with 25 µg/mL propidium iodide (Sigma-Aldrich). The cell cycle distribution was evaluated by flow cytometry, counting >10,000 cells per sample.

### Immunofluorescence

Cells were plated on chamber slides, fixed with acetone/methanol. (1∶1), and non-specific binding sites were blocked with 3% (m/vol) BSA. Fixed cells were incubated with appropriate primary antibodies for 60 minutes in PBS containing 0.1% (m/vol) BSA. Indirect immunofluorescence was performed by incubation with FITC- or Alexa 555–conjugated secondary antibodies (Zymed and Molecular Probes; Invitrogen and Life Technologies, Grand Island, N.Y., USA, respectively). Nuclei were visualized by DAPI (Roche Diagnostics, Florham Park, N.J., USA). Immunofluorescence was quantified by counting the mean number of immunostained nuclei per high-power field. Results are presented as the average of at least 3 counts.

### Nuclear and cytoplasmic protein fraction isolation

Cells cultured in 6cm dishes were washed twice with PBS. The PBS was discarded and 250 µl lysis buffer (10 mM Hepes-NaOH, pH 7.9, 10 mM KCl, 1.5 mM MgCl_2_, and 0.5 mM β-mercaptoethanol) supplemented with protease inhibitor mixture and phosphatase inhibitors was added into each 6 cm dish, the cells were scraped into the lysis buffer and vortexed, then incubated on ice for 15–20 min. 4–5 µl 10% NP-40 was added and the sample was vortexed and put on ice for 2 min. Then centrifuged at 16000 g for 10–15 min. The supernatant contained the cytoplasmic proteins. The pellet was washed twice with ice-cold PBS (200 µl/each), then 60–80 µl nuclei lysis buffer (10 mM Tris-HCl, pH 7.6, 420 mM NaCl, 0.5% Nonidet P-40, and 1 mM DTT, 1 mM PMSF, 2 mM MgCl_2_ plus protease and phosphatase inhibitors) was added to the pellet, which was dispersed using a pipette tip and put on ice for 20 min. The suspension was centrifuged at 16000 g for 10–15 min, the resulting extract contained nuclear proteins. Finally, lower salt buffer (10 mM Tris-HCl, pH 7.6, 1 mM DTT, 1 mM PMSF, 2 mM MgCl_2_ plus the protease and phosphatase inhibitors) was added to adjust the concentration of NaCl to 150 mM to allow for downstream SDS-PAGE separation. Membrane protein extraction buffer (ProteoJET Membrane Protein Extraction Kit #K0321) was then added to the pellet and the mixture was incubated for 30 min at 4°C with constant shaking at 450 rpm. The membrane protein extract was collected in a tube and centrifuged at 16000 g for 15 min at 4°C. The supernatant containing the membrane protein fraction was transferred into a new tube for later analysis.

### Western blot analysis

Cells were harvested and lysed in extraction buffer (20 mM Tris-Cl, 150 mM NaCl, 1 mM EDTA, and 1 mM EGTA, 1% m/vol Triton X-100, supplemented with Complete Proteinase Inhibitor Cocktail [Merck Millipore, Darmstadt, Germany]). Protein extracts were clarified by centrifugation (6,000 g) at 4°C. Protein concentrations were determined using the Bradford method, and 25 µg total protein were separated on 10% SDS-polyacrylamide gels. Separated proteins were transferred onto nitrocellulose membranes (Life Technologies). Membranes were blocked with 5% nonfat dry milk in TBS and incubated with primary antibodies. After washing, membranes were incubated with secondary, HRP-linked antibodies (Pierce, Rockford, Ill., USA). Proteins were visualized by enhanced chemiluminescence and autoradiography (ECL; Amersham Biosciences, GE Healthcare, Pittsburgh, Penn., USA). ImageJ was used to analyze the density of the bands.

### Cell counting kit-8 assays

The cell counting kit-8 (CCK-8) assays were performed following the manufacturer's protocol. Ten µl of various concentrations of anti-WISP-1 antibody were added. The plate was incubated for an appropriate length of time (e.g., 6, 12, 24, 48 or 72 hours). Results were measured by absorbance at 450 nm using an ELx800 microplate reader (BioTek Instruments Inc.,Winooski,Vermont,USA).

### *In vivo* tumorigenicity assays

All the animal protocols in this study were conducted in accordance with the institutional animal welfare guidelines. The use of mice was approved by the ethics committee of the first affiliated hospital of Wenzhou medical university. All mice were sacrificed by cervical dislocation. To develop xenograft tumors, cultured KYSE-410 and KYSE-410R cells were harvested by exposure to trypsin-EDTA, washed with PBS, and implanted into the right flanks of 4-week-old male NOD/SCID mice, weight 6.1–7.2 g (1.0×10^5^ cells for both KYSE-410 and KYSE-410R). When all the mice developed tumors (3 weeks after injection), they were subjected to fractionated irradiation (20 Gy in total, 2 Gy per fraction) and/or treatment with 2 µg/ml recombinant WISP-1 protein or 4 µg/ml anti-WISP-1 antibody. Each group included 5 mice. WISP-1 and anti-WISP-1 were administered intravenously via the caudal vein. Absolute tumor volume (mm^3^) was calculated by the following formula: V(mm^3^) = A(mm)×B(mm)^2^/2, where A and B were the longest and widest diameter of the tumor, respectively, measured every week using calipers. The tumor volume of the first measurement was normalized to 1, and relative tumor volume was calculated as observed measurement versus corresponding first measurement. Data is presented as fold-change in volume, log_2_ (observed/corresponding first).

### Clinical specimens

Biopsy samples were obtained from 10 esophageal carcinoma patients before and after radiotherapy (mean age, 57±6 years; 3 females, 7 males). Samples were snap-frozen in liquid nitrogen immediately after their explants.

### Immunohistochemistry

Tissues were fixed in 10% formalin, embedded in paraffin, and then cut into 4-µm sections. After deparaffinization, rehydration, and inactivation of endogenous peroxidase by hydrogen peroxide, the sections were blocked with normal donkey serum, and then incubated with WISP-1 antibody (1∶100; ab10737, Abcam, Cambridge, UK) overnight at 4 °C. After washing twice with PBS, the sections were then incubated with horseradish peroxidase-conjugated donkey anti-goat IgG (Santa Cruz Biotechnology, Santa Cruz, CA, USA) at 37 °C for 1 h. After washing, the sections were visualized with diaminobenzidine, counterstained with hematoxylin, and photographed using a light microscope at 100 or 400 magnification. Normal goat serum and secondary antibody alone were used as negative controls.

### Statistics

Statistical analysis was conducted using the student t-test or the Mann-Whitney U-test and the Fisher's exact two-tailed test for western blot analysis with SPSS (version 17.0, SPSS Inc., Chicago, USA). All results are presented as mean ± SD. A probability level of 0.05 was considered statistically significance.

## Results

### Elevated expression of WISP-1 in established radioresistant cancer cells

**Figure 1:**
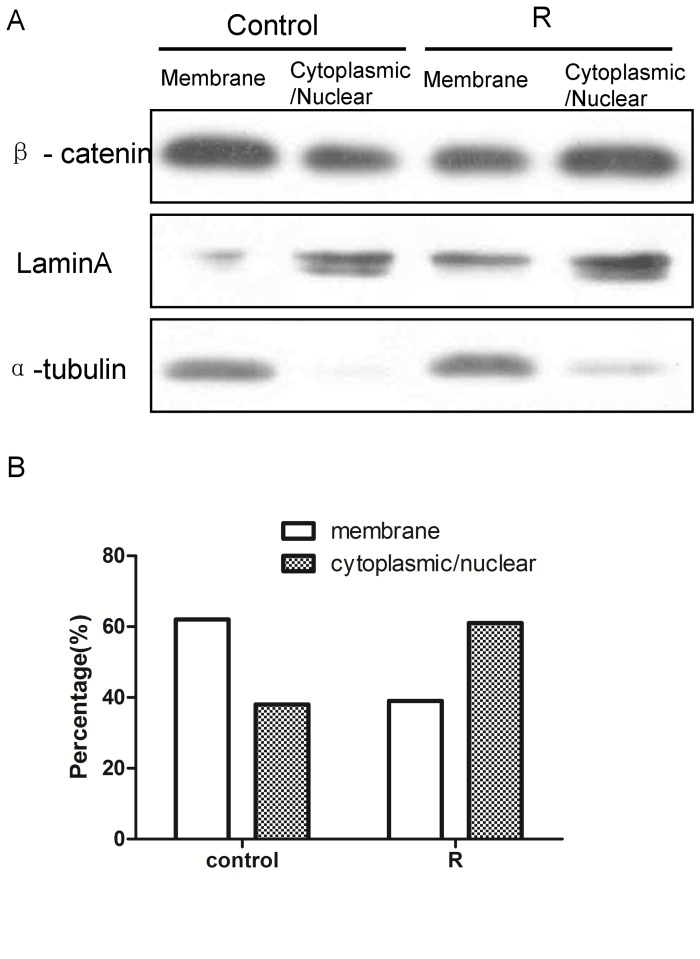
Translocation of β-catenin. Western blotting analysis showed β-catenin in the membrane and cytoplasmic/nuclear cell fractions. α-tubulin was the loading control for the membrane fraction. Lamin A was the loading control for the cytoplasmic/nuclear fraction. Data are representative of at least 3 independent experiments. The graph shows the relative percentage of β-catenin in the corresponding location. Approximately 100 cells were evaluated, the odds ratio for cytoplasmic and or nuclear translocation of β-catenin in radioresistant cells versus their parents was 2.552 (95% CI: 1.443 to 4.512) and the Fisher's exact two-tailed was *P* = 0.0018.

To explore the molecular mechanisms involved in radioresistance, radioresistant esophageal cancer cell lines were established by applying repeated 2-Gy FIR using methodology described by Zhang et al [4]. The clonogenic survival curves confirmed that KYSE-410R and TE-1R were more radioresistant than parental cells (Figure S1). Phosphorylation of β-catenin at Ser33 and Ser37 was decreased in radioresistant cells (Figure S2A); this can promote β-catenin stabilization and accumulation [12]. The results of immunofluorescence staining further confirmed that the stability of β-catenin and β-catenin nuclear translocation were promoted more in the radioresistant cancer cells than in their parents (Figure S2B.). These results indicated constitutive activation of the β-catenin signal in radioresistant esophageal carcinoma cells. To further evaluate the translocation of β-catenin, we confirmed the level of β-catenin contained within the nuclear and cytoplasmic cell fractions. To semi-quantitatively evaluate the translocation of β-catenin, we assumed that the relative expression level detected by western blotting was proportional to the number of the cells expressing β-catenin. Therefore, the difference could be analyzed by Fisher's exact two-tailed test. The mean percentage of cells with β-catenin cytoplasmic and or nuclear expression was 61% for radioresistant cells and 38% for their parents (Figure 1). Evaluation was undertaken for approximately 100 cells, the odds ratio for cytoplasmic and or nuclear translocation of β-catenin in radioresistant cells versus their parents was 2.552 (95% CI: 1.443 to 4.512) and the Fisher's exact two-tailed was *P* = 0.0018.

Additionally, Western blot analysis identified phosphorylated GSK3β (p-Ser9 GSK3β), total GSK3β, phosphorylated β-catenin (p-Ser33/Ser37/Thr41β-catenin and p-Ser45 β-catenin) and total β-catenin in KYSE-410 cells when treated with irradiation. The data showed the p-Ser33/Ser37/Thr41 β-catenin levels relative to total β-catenin levels in 5 fraction irradiated cells were decreased 2.8 fold compared to the non-irradiation control. The p-Ser9 GSK3β levels relative to total GSK3β levels in 5 fraction irradiated cells were increased 1.2 fold compared to the non-irradiation control (Figure S2C.).

To elucidate whether Wnt/β-catenin activation was involved, we sought to quantify the mRNA expression of canonical Wnt/β-catenin signaling components in both parental and radioresistant cell lines (Figure S2D). The results showed that compared to KYSE-410 cells, the expression of Wnt ligands Wnt1, β-catenin, the common Wnt receptors frizzled 1–4 (Fzd1- 4) and the intracellular signal transducers Gsk3b was upregulated in KYSE-410R cells.

**Figure 2:**
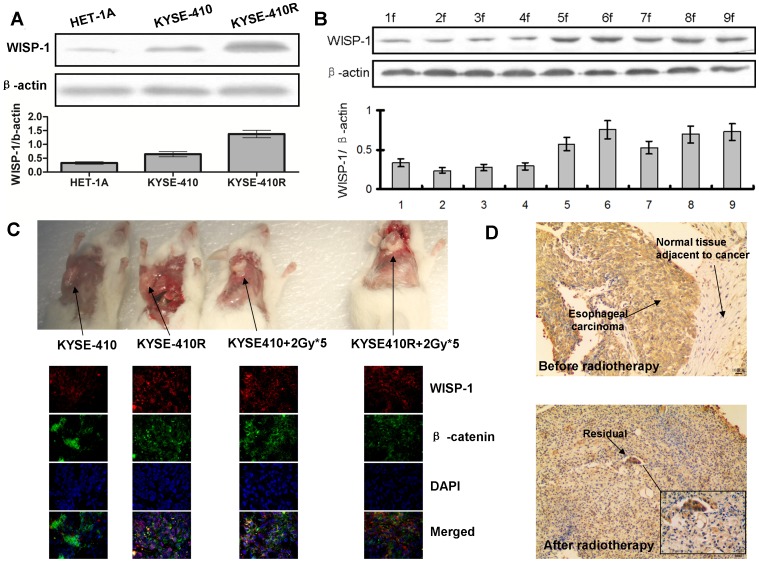
FIR-induced WISP-1 over-expression in esophageal cancer cells. A. Western blotting analysis showed WISP-1 in the radioresistant cells was overexpressed, compared to their parents and normal esophageal epithelial cells. β-actin was the loading control. Data are representative of at least 3 independent experiments. The graph shows the mean band intensity (±SD). B. WISP-1 protein expression was determined in total protein lysates from KYSE-410 cells during FIR using Western blotting analysis. β-actin was the loading control. Data are representative of at least 3 independent experiments. 1, 2, 3, 4, 5, 6, 7, 8, 9f represented first, second, third, fourth, fifth, sixth, seventh, eight, and ninth fraction (2 Gy) of irradiation. The graph shows the mean values (±SD) of relative expression of WISP-1 versus β-actin. C. The increased expression of β-catenin and WISP-1 in radioresistant KYSE-410R cells was then further confirmed by immunofluorescence staining in vivo with tumor tissues from mice. D. WISP-1 protein expression was assessed using immunohistochemical staining on specimens from clinical patients before (upper panel, ×100) and after (bottom panel, ×100, ×400) radiotherapy.

Screening for potential downstream targets of β-catenin revealed that expression of WISP-1 in radioresistant cells was elevated compared to parental cells (1.4 fold) and to normal esophageal epithelial cells (2.7 fold) (Figure 2A). Increased WISP-1 expression was induced by FIR; WISP-1 expression reached a stable level of expression during FIR, after 5 fractions (2Gy/fraction) (Figure 2B). The increased expression of β-catenin and WISP-1 in radioresistant cells was then further confirmed by immunofluorescence staining in vivo with tumor tissues from mice (Figure 2C). We also confirmed the level of WISP-1 in clinical specimens by using immunohistochemistry (Figure 2D). This showed that the esophageal cancer cells were positively stained for WISP-1 and the normal tissue cells adjacent to cancer were weakly stained. The residual cancer cells after radiotherapy were more positively stained as along with the surrounding stromal components.

### WISP-1 in medium promoted radioresistance of esophageal carcinoma cells

**Figure 3:**
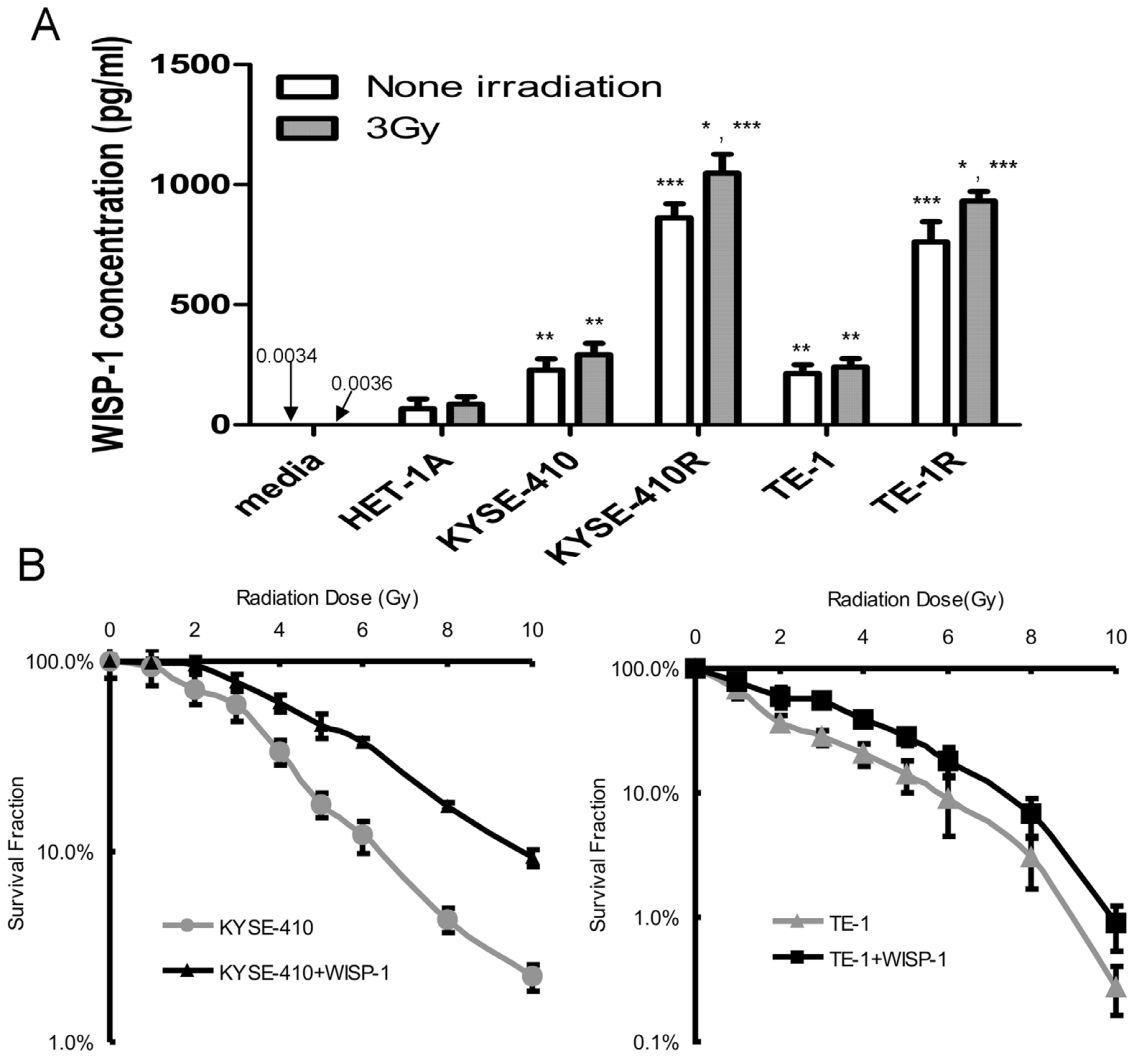
WISP-1 in medium promoted radioresistance of esophageal carcinoma cells. A. ELISA assays (in triplicate) for WISP-1 levels in the conditioned medium (*P<0.05 when compared with non irradiated, **P<0.01 when compared with media alone or HET-1A, ***P<0.001 when compared with media alone or HET-1A or their parents). B. Recombinant WISP-1 stimulation elevated radioresistance in esophageal cancer cells. Clonogenic survival in recombinant WISP-1 protein (2 µg/ml) treated KYSE-410 and TE-1 cells after irradiation. The data points show mean survival fraction from 5 individual experiments (±SD).

As it has been suggested that WISP-1 is an extracellular secreted protein [13], we therefore investigated the levels of secreted protein in the culture medium (Figure 3A). Then, the effect of recombinant WISP-1 on KYSE-410 and TE-1 was assessed. The results showed that addition of WISP-1 to medium resulted in significantly increased radioresistance (*P* = 0.017 and 0.031, in KYSE-410 and TE-1, respectively) in colony forming assays (Figure 3B). To confirm the result of recombinant protein used, we replaced the recombinant protein with conditioned medium obtained after radiation treatment that should have high level secret WISP-1 for survival experiments. The colony forming assays showed that culturing with conditioned medium also elevated radioresistance in normal esophageal cancer cells (Figure S3).

In order to test whether WISP-1 modulated cancer cell radioresistance by altering cell-cycle distribution, cells were subjected to cell-cycle analysis by flowcytometry, 24 hours after WISP-1 treatment. The results showed that the WISP-1 treatment did not significantly alter cell-cycle distribution (Figure S4) compared to control cells.

In order to investigate the mitotic state of irradiated cells and WISP-1 treated irradiated cells, immunofluorescence with anti β-tubulin staining of nuclear microtubules was performed. The results showed the microtubules of irradiated cells were disturbed, and multiple poles had formed in the cell nuclei (Figure S5). In contrast, treatment with recombinant WISP-1 caused irradiated cells to progress through mitosis with enhanced assembly of nuclear microtubules. There was less disturbance of the mitotic state and fewer multiple poles were formed in WISP-1 treated irradiated cells compared to irradiated cells that were not exposed to WISP-1. These data indicate that extracellular WISP-1 protein plays an important role in esophageal cancer cells and contributes to the development of radioresistance.

### Depletion of extracellular WISP-1 protein attenuated the radioresistance of established radioresistant cancer cells

**Figure 4:**
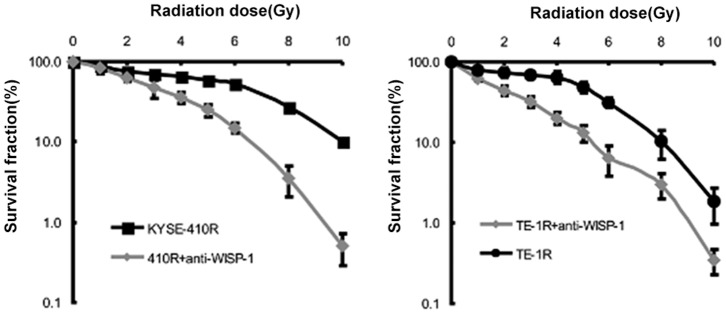
Depletion of extracellular WISP-1 protein attenuated the radioresistance of established radioresistant cancer cells. Clonogenic survival in anti-WISP-1 antibody (4 µg/ml) treated radioresistant esophageal cancer cells after irradiation. The data points show mean survival fraction from 5 individual experiments (±SD).

As radioresistant cells secreted greater amounts of WISP-1 compared to parental cells, we sought to deplete WISP-1 in eospohageal cancer cells using anti-WISP-1 antibodies; these are known to be effective in neutralizing extracellular WISP-1 activity both *in vitro* and *in vivo* [14]. The results of colony forming assays showed anti-WISP-1 treatment significantly (*P*<0.001 and *P* = 0.006, respectively) attenuated radioresistance in KYSE-410R and TE-1R cells (Figure 4).

### Depletion of extracellular WISP-1 protein promotes mitotic catastrophe in radioresistant cancer cells

**Figure 5:**
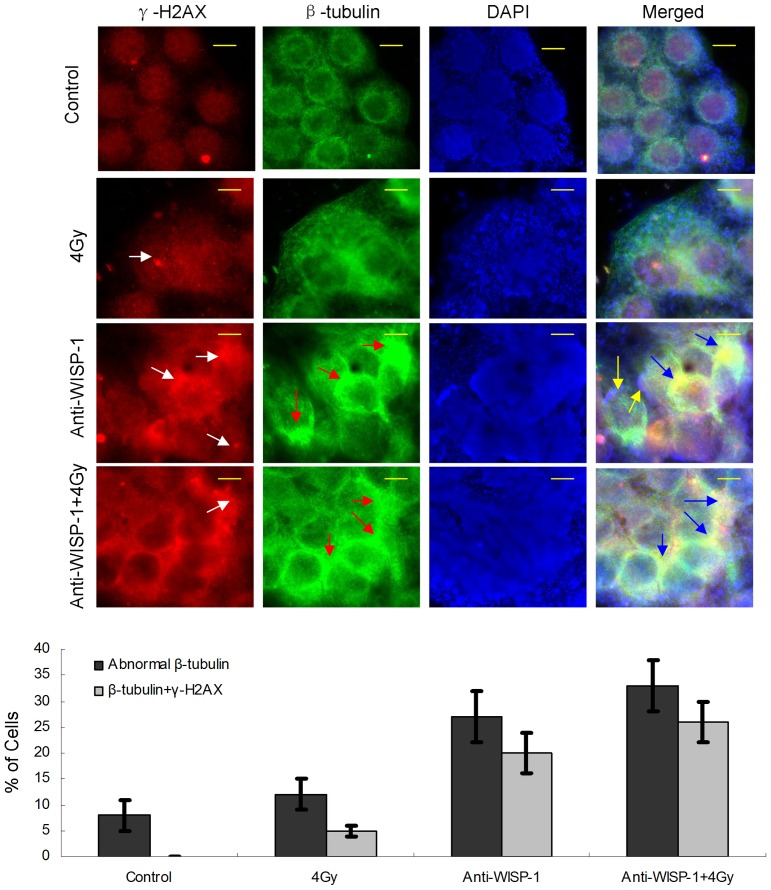
Depletion of extracellular WISP-1 protein promoted mitotic catastrophe in radioresistant cancer cells. KYSE-410R cells were treated with anti-WISP-1 antibody (4 µg/ml), 4 Gy of radiation, or a combination. Immunofluorescence staining for nuclei (DAPI, blue), γ-H2AX (red) and β-tubulin (green). Scale bars, 10 µm. White arrows indicate γ-H2AX foci; red arrows indicate assembly of abnormal microtubules; blue arrows indicate atypical β-tubulin staining (green) co-stained withγ-H2AX (red). Yellow arrows indicate lagging chromosomal material. Data are representative of at least 3 independent experiments. Immunofluorescence was quantified by counting the mean number of nuclei per high-power field. The graph shows the quantification of nuclei positive for abnormal β-tubulin staining and β-tubulin and γ-H2AX co-staining. The graph shows the means ± SE of three independent experiments. For every experiment at least 200 nuclei were counted per treatment condition.

Following anti-WISP-1 treatment, nuclear staining of KYSE-410R using DAPI showed the formation of large cells with lobulated micronuclei, and/or lagging chromosomal material, identified as mitotic catastrophe [15], [16], [17]. In order to confirm the extent of mitotic catastrophe, we performed immunofluorescence with β-tubulin and γ-H2AX antibodies. Figure 5 shows KYSE-410R displaying signs of mitotic catastrophe with abnormal staining of β-tubulin, γ-H2AX and DNA, 24 hours after anti-WISP-1 treatment. Immunofluorescence staining for β-tubulin showed that microtubules in radioresistant cells were disturbed by anti-WISP-1 treatment; the disturbance was aggravated when combined with 4 Gy irradiation (based on a pilot experiment). Treatment-induced mitotic catastrophe was further evident when cells were co-stained with tubulin and γ-H2AX. The percentage of β-tubulin and γ-H2AX co-stain in nucleus was significantly increased in the presence of anti-WISP-1.

### Anti-WISP-1 treatment specifically targets FIR established radioresistant cancer cells

**Figure 6:**
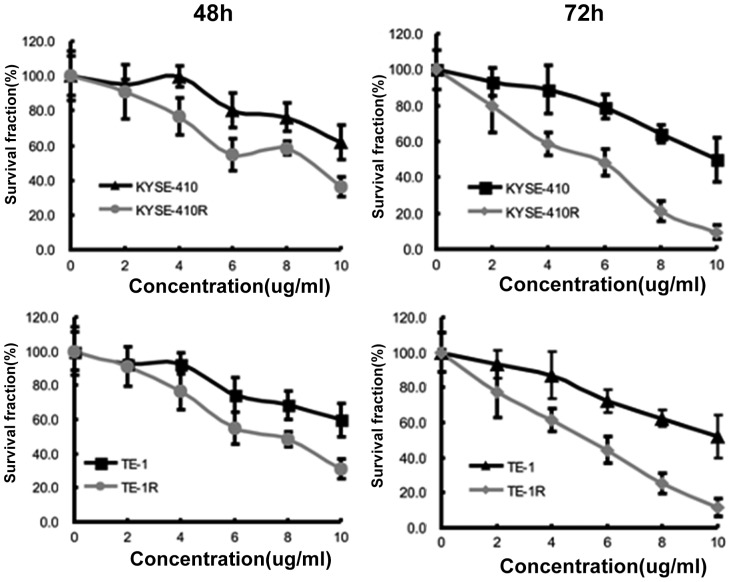
WISP-1 antagonism specifically targeted FIR induced radioresistant cancer cells. Graphs show the results of CCK-8 assays 48 hours (left panel) and 72 hours (right panel) after anti-WISP-1 treatment of KYSE-410 and KYSE-410R cells. Data are presented as means ± SD (n = 12). DMSO controls validated these results (data not shown).

As anti-WISP-1 treatment could directly induce mitotic catastrophe in radioresistant cancer cells established by FIR, we sought to confirm whether this was a specific therapeutic effect or general cell toxicity. We performed CCK-8 assays following anti-WISP-1 treatment of KYSE-410 and KYSE-410R cells. The results showed that anti-WISP-1 treatment resulted in more death in radioresistant cells compared to parental cells. The differences were not significant 24 hours after anti-WISP-1 treatment (*P* = 0.073, data not shown), but became significant 48 hours and 72 hours after treatment (*P* = 0.02, *P* = 0.009, Figure 6; respectively).

Colony-forming assays indicated that anti-WISP-1 pretreatment of KYSE-410 cells did not significantly enhance their radiosensitivity (data not shown), nor were the effects of anti-WISP-1 in these cells substantially different from those elicited in radioresistant cancer cells.

### Role of WISP-1 mediated radioresistance *in vivo*

**Figure 7:**
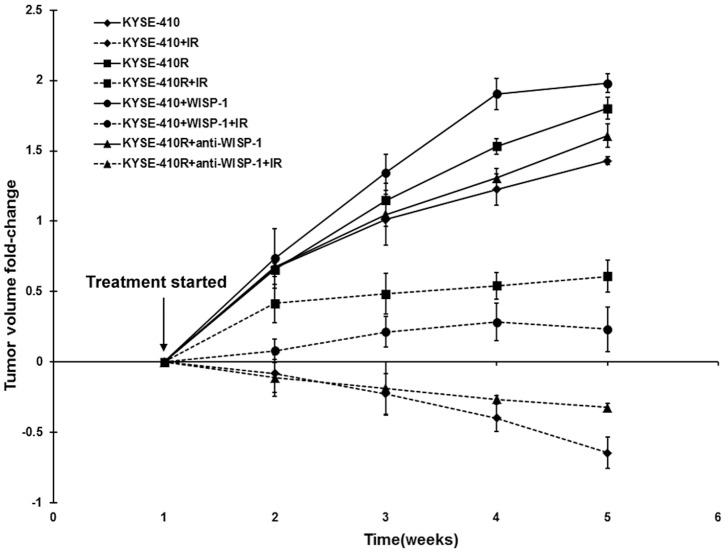
The role of WISP-1 mediated radioresistance in vivo. NOD-SCID mice bearing KYSE-410 or KYSE-410R tumors were treated with radiotherapy, 2 µg/ml recombinant WISP-1 protein or 4 µg/ml anti-WISP-1 antibody, or a combination. The change in tumor volume was calculated as described in “Materials and Methods”. Data are presented as means ± SD (n = 6). BSA controls validated these results (data not shown).

In order to validate the therapeutic effect of anti-WISP-1 treatment *in vivo*, we developed a xenograft tumor model in NOD/SCID mice. Figure 7 shows the relative tumor volumes in 8 groups of mice. These data show that KYSE-410R cells grew more rapidly than KYSE-410 cells, and the growth of KYSE-410 cells stimulated with 2 µg/ml recombinant WISP-1 protein was also accelerated. The growth of KYSE-410R cells treated with 4 µg/ml anti-WISP-1 was reduced compared to untreated KYSE-410R, and the growth rate was comparable with untreated KYSE-410 cells. When irradiation was applied, the KYSE-410R cells showed significantly more resistance than KYSE-410 cells (*P* = 0.003). Recombinant WISP-1 protein stimulation significantly increased the radioresistance of KYSE-410 cells *P* = 0.008), and anti-WISP-1 treatment significantly reversed the radioresistance of KYSE-410R cells (*P* = 0.005).

The *in vivo* results also showed that anti-WISP-1 treatment cannot enhance radiosensitivity in KYSE-410 cells (data not shown), which is in accordance with our *in vitro* data.

## Discussion

Understanding radioresistance in cancer cells is important for developing therapeutic strategies. Zhang et al. showed that radioresistant esophageal cancer cells can be established by FIR [4]; therefore, it is possible that current fractionated radiotherapy leaves residual tumors and the risk of recurrence. While previous work indicates that irradiation-induced aberrant activation of the Wnt/β-catenin pathway is linked to the development of radioresistance in several tumors [4], [6], [7], the details remained elusive. Our results showed that the expression of WISP-1 was increased during FIR until it reached high and stable levels. Our study confirms that WISP-1 plays an important role in FIR-induced radioresistance in esophageal carcinoma cells, as the expression of WISP-1 correlated with the development of radioresistance in these cells, and radioresitance was induced by the presence of recombinant WISP-1 in medium.

We observed that extracellular WISP-1 protein promoted mitosis, which may contribute to the development of radioresistance during FIR. Interestingly, depletion of extracellular WISP-1 protein in established radioresistant cells directly induced a mass of γ-H2AX foci and obvious mitotic catastrophe, but depletion of extracellular WISP-1 protein in normal cancer cells did not have this effect. This result indicates that radioresistant cancer cells may exist in a sublethal state, and that FIR-induced damage in cancer cells does not result in cell death. Indeed, extracellular WISP-1 protein may provide important support for their survival.

Mitotic catastrophe is a well-documented phenomenon that occurs in tumor cells treated with DNA damaging agents [18], [19], [20], [21], [22]. It is thought to be the primary means of tumor cell death resulting from DNA-damaging therapeutic agents [18]. A number of DNA damage response proteins are known to be associated with the stabilization of the centrosome and with spindle construction, as well as being involved in the negative regulation of mitotic cell death in response to DNA damage. These DNA damage response proteins include Chk2, which is known to be necessary for centrosome stability and may protect against irradiation induced mitotic catastrophe via phosphorylation of BRCA1/T988 [23]. Shang et al. recently showed Chk2 is a negative regulator of mitotic catastrophe, as they demonstrated that DNA damage resulted in inactivation of DNA-PKcs, spindle disruption and mitotic catastrophe with attenuated Chk2 phosphorylation [24]. Other studies indicate that Chk2 may enhance DNA repair by modulating p53 [25], [26], [27], [28], [29]. Interestingly, we found that irradiation in the presence of high concentrations of extracellular WISP-1 protein did not affect the phosphorylation of Chk2 in esophageal cancer cells (Figure S6). It is possible that the presence of WISP-1 inhibited the normal response of Chk2 to irradiation. We suspect that activation of Chk2 in the presence of WISP-1 maintains centrosome stability and prevents significant spindle disruption during mitosis. This may promote DNA repair and ensure the survival of cancer cells during FIR. However, the role of Chk2 in this process remains unclear; therefore, the details of how WISP-1 mediates such complex processes warrant further study.

## Conclusion

In conclusion, we found that overexpression of WISP-1 protein contributed to the development of radioresistance in esophageal cancer cells during FIR. Depletion of extracellular WISP-1 protein attenuated the radioresistance of established radioresistant cancer cells and directly induced mitotic catastrophe. WISP-1 may be a candidate therapeutic target when treating recurrent esophageal carcinoma after radiotherapy.

## Acknowledgments

We thank Professor Yunsheng Xu and XiangWu Zheng from the First Hospital Wenzhou Medical University, for general support.

## Supporting Information

Figure S1 Clonogenic survival in normal esophageal cancer cells (KYSE-410 and TE-1) and FIR induced radioresistant esophageal cancer cells (KYSE-410R and TE-1R) after irradiation. The data points show the mean survival fraction from 5 individual experiments (±SD).
(TIF)

Figure S2 A. Protein expression of β-catenin detected with normal or phospho-specific (Ser33/37) antibodies in total protein lysates from KYSE-410 or KYSE-410R cells using Western blotting analysis. β-actin was the loading control. Data are representative of at least 3 independent experiments. Control = KYSE-410, R = KYSE-410R. B. β-catenin expression and nuclear translocation comparison between KYSE-410 and KYSE-410R were assessed by immunofluorescence detection of β-catenin (Green). Nuclei were visualized by DAPI staining (blue), Scale bars (red), 10 µm. Data are representative of at least 3 independent experiments. The white arrow indicated the β-catenin nuclear translocation. C. Western blot analysis detected phosphorylated GSK3β (p-Ser9 GSK3β), total GSK3β, phosphorylated β-catenin (p-Ser33/Ser37/Thr41 β-catenin and p-Ser45 β-catenin) and total β-catenin in KYSE-410 cells when treated with irradiation. GAPDH was used as a load control. Data are representative of at least 2 independent experiments. D. The mRNA expression of the selected genes in the Wnt/β-catenin signaling pathway (including Wnt1, β-catenin, Fzd1-4, and Gsk3β) was measured by qRT-PCR in KYSE-410R versus KYSE-410. The experiments were repeated for 5 times. The data were presented as mean ± SD (n = 5), and the results of KYSE-410R cells were normalized to KYSE-410 cells.
(TIF)

Figure S3 Conditioned medium culture elevated radioresistance in esophageal cancer cells. Clonogenic survival in conditioned medium cultured with KYSE-410 and TE-1 cells after irradiation. The data points show mean survival fraction from 5 individual experiments (±SD).
(TIF)

Figure S4 KYSE-410 cells were treated with recombinant WISP-1 (2 µg/ml), 4 Gy of radiation, or a combination. Cell cycle distributions of the indicated populations were achieved through propidium iodide DNA staining and flow cytometry analysis. The data were analyzed with student t-test. G1: Blue; S: Striped Yellow; G2:Red. Data shown are representative of two independent experiments.
(TIF)

Figure S5 KYSE-410 cells were treated with recombinant WISP-1 (2 µg/ml), 4 Gy of radiation or a combination. Immunofluorescence staining for nuclei (DAPI, blue) and β-tubulin (green). Scale bars, 40 µm. Red arrows indicate disturbed microtubule distribution with multiple poles formed in the nucleus; white arrows indicate normal microtubule distribution during successful mitosis. Data are representative of at least 3 independent experiments.
(TIF)

Figure S6 Cells were treated with recombinant WISP-1 alone (2 µg/ml), 4 Gy of radiation alone, or the combination of both. Protein expressions of Chk2 with normal or phospho-specific antibodies (thr68), ATM and DNA-PKcs were determined in total protein lysates from indicated populations using Western blotting analysis. β-actin was the loading control. Data are representative of at least 3 independent experiments.
(TIF)
