# Supplementary figures and images for: WISP-1 Contributes to Fractionated Irradiation-Induced Radioresistance in Esophageal Carcinoma Cell Lines and Mice
Source: PLoS One. 2014 Apr 11;9(4):e94751. doi: 10.1371/journal.pone.0094751 (PMC3984255; doi:10.1371/journal.pone.0094751)

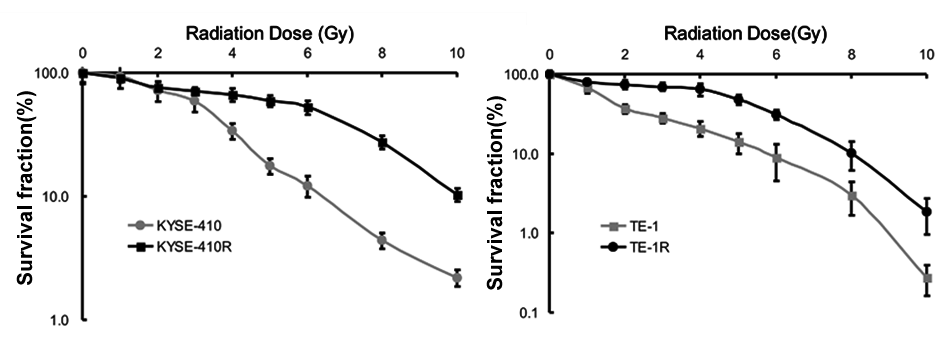

Supplement: Figure S1 — Clonogenic survival in normal esophageal cancer cells (KYSE-410 and TE-1) and FIR induced radioresistant esophageal cancer cells (KYSE-410R and TE-1R) after irradiation. The data points show the mean survival fraction from 5 individual experiments (±SD). (TIF) [file pone.0094751.s001.tif]

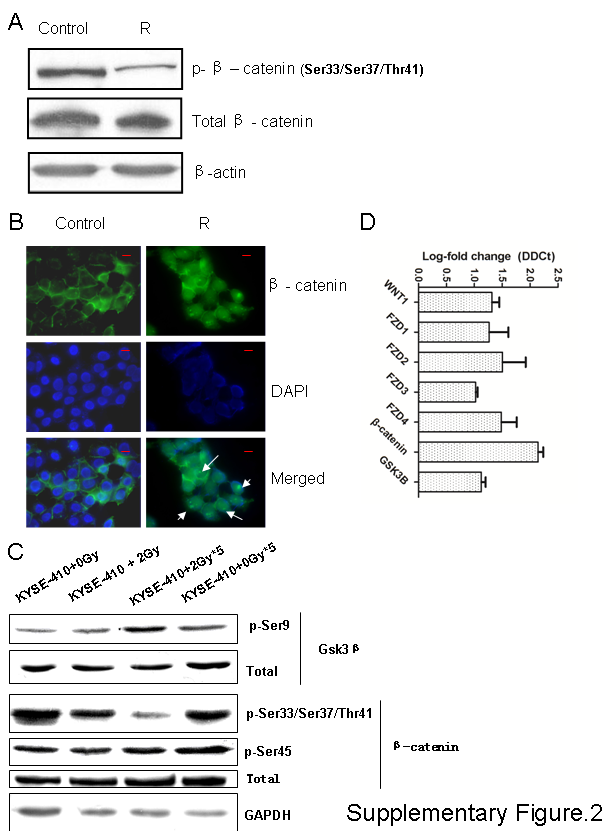

Supplement: Figure S2 — A. Protein expression of β-catenin detected with normal or phospho-specific (Ser33/37) antibodies in total protein lysates from KYSE-410 or KYSE-410R cells using Western blotting analysis. β-actin was the loading control. Data are representative of at least 3 independent experiments. Control = KYSE-410, R = KYSE-410R. B. β-catenin expression and nuclear translocation comparison between KYSE-410 and KYSE-410R were assessed by immunofluorescence detection of β-catenin (Green). Nuclei were visualized by DAPI staining (blue), Scale bars (red), 10 µm. Data are representative of at least 3 independent experiments. The white arrow indicated the β-catenin nuclear translocation. C. Western blot analysis detected phosphorylated GSK3β (p-Ser9 GSK3β), total GSK3β, phosphorylated β-catenin (p-Ser33/Ser37/Thr41 β-catenin and p-Ser45 β-catenin) and total β-catenin in KYSE-410 cells when treated with irradiation. GAPDH was used as a load control. Data are representative of at least 2 independent experiments. D. The mRNA expression of the selected genes in the Wnt/β-catenin signaling pathway (including Wnt1, β-catenin, Fzd1-4, and Gsk3β) was measured by qRT-PCR in KYSE-410R versus KYSE-410. The experiments were repeated for 5 times. The data were presented as mean ± SD (n = 5), and the results of KYSE-410R cells were normalized to KYSE-410 cells. (TIF) [file pone.0094751.s002.tif]

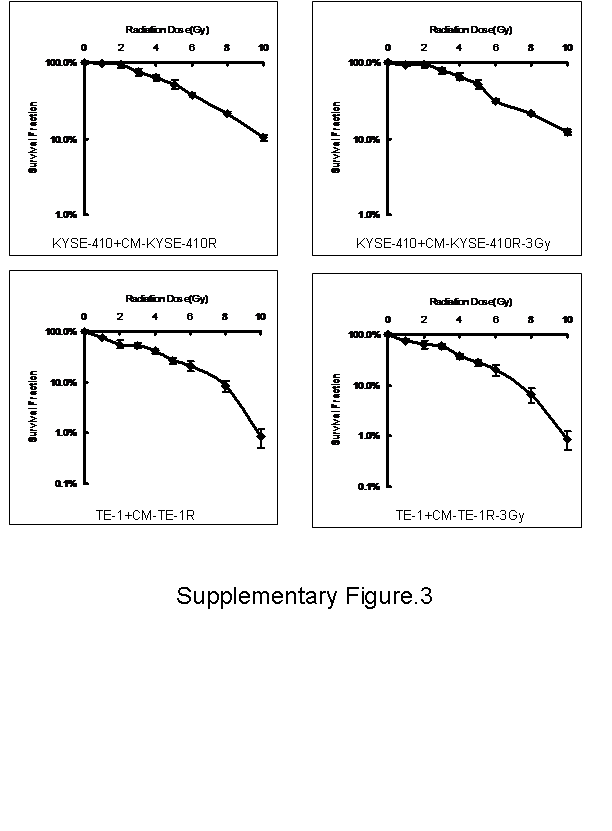

Supplement: Figure S3 — Conditioned medium culture elevated radioresistance in esophageal cancer cells. Clonogenic survival in conditioned medium cultured with KYSE-410 and TE-1 cells after irradiation. The data points show mean survival fraction from 5 individual experiments (±SD). (TIF) [file pone.0094751.s003.tif]

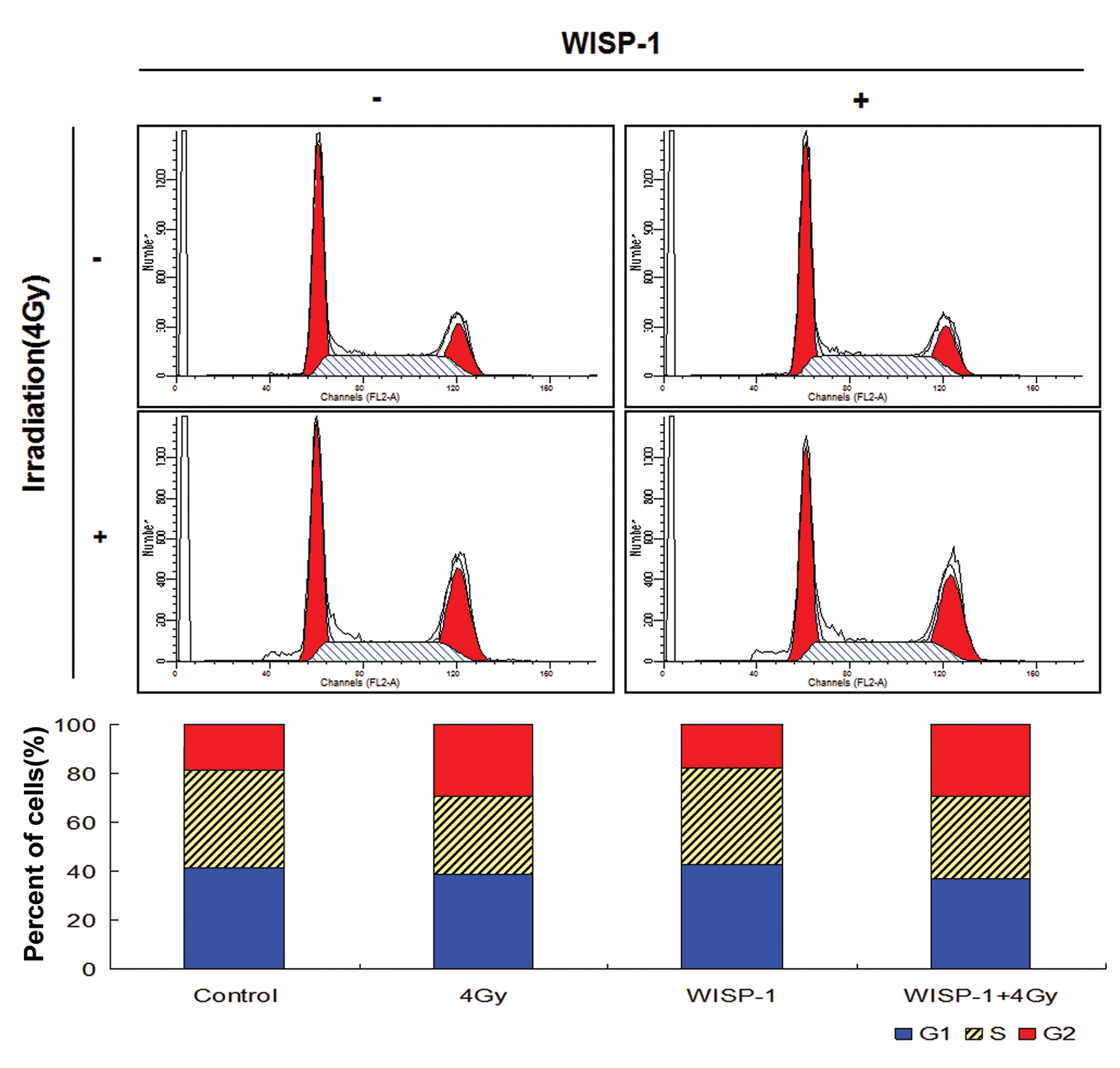

Supplement: Figure S4 — KYSE-410 cells were treated with recombinant WISP-1 (2 µg/ml), 4 Gy of radiation, or a combination. Cell cycle distributions of the indicated populations were achieved through propidium iodide DNA staining and flow cytometry analysis. The data were analyzed with student t-test. G1: Blue; S: Striped Yellow; G2:Red. Data shown are representative of two independent experiments. (TIF) [file pone.0094751.s004.tif]

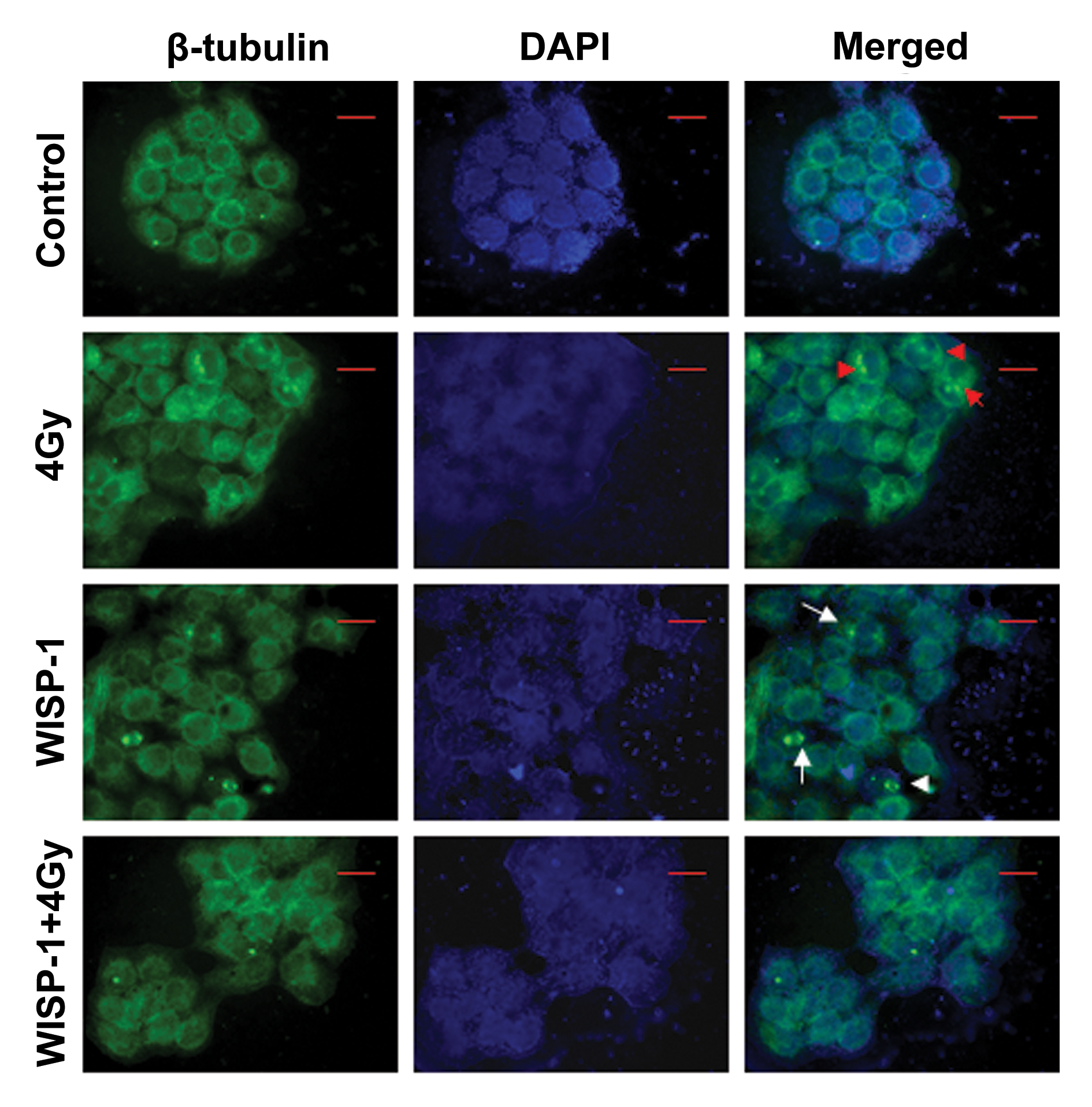

Supplement: Figure S5 — KYSE-410 cells were treated with recombinant WISP-1 (2 µg/ml), 4 Gy of radiation or a combination. Immunofluorescence staining for nuclei (DAPI, blue) and β-tubulin (green). Scale bars, 40 µm. Red arrows indicate disturbed microtubule distribution with multiple poles formed in the nucleus; white arrows indicate normal microtubule distribution during successful mitosis. Data are representative of at least 3 independent experiments. (TIF) [file pone.0094751.s005.tif]

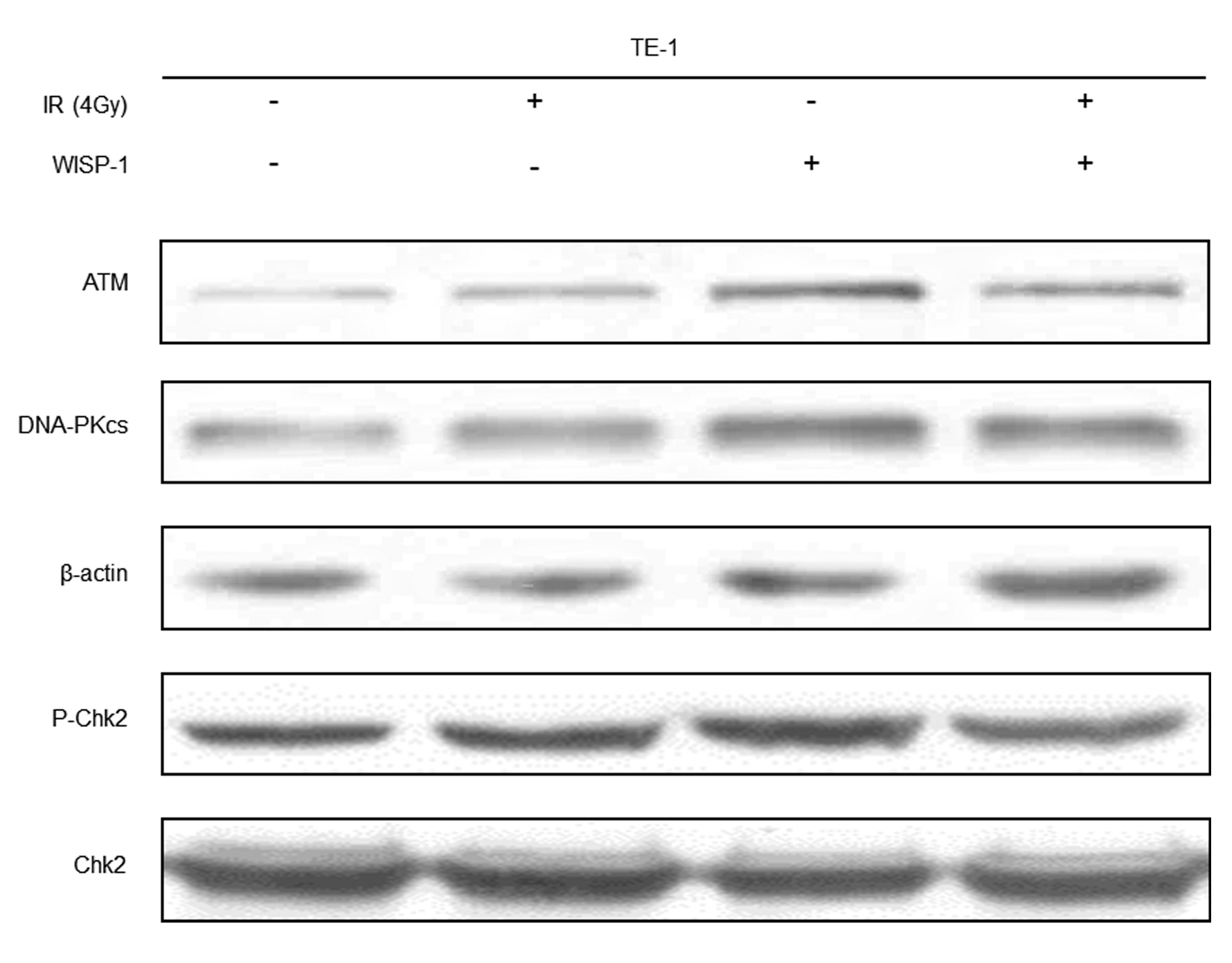

Supplement: Figure S6 — Cells were treated with recombinant WISP-1 alone (2 µg/ml), 4 Gy of radiation alone, or the combination of both. Protein expressions of Chk2 with normal or phospho-specific antibodies (thr68), ATM and DNA-PKcs were determined in total protein lysates from indicated populations using Western blotting analysis. β-actin was the loading control. Data are representative of at least 3 independent experiments. (TIF) [file pone.0094751.s006.tif]
